# Application of New Approach Methodologies to Improve Oral Biopharmaceutic Assessments

**DOI:** 10.3390/pharmaceutics18050552

**Published:** 2026-04-30

**Authors:** Mauricio A. García, Miguel Ángel Cabrera-Pérez, Pablo M. González, Alexis Aceituno, Daniel Hachim

**Affiliations:** 1 Escuela de Química y Farmacia, Facultad de Química y de Farmacia, Pontificia Universidad Católica de Chile, Santiago 7820436, Chile; magarci3@uc.cl; 2 Departamento de Ciencias Farmacéuticas, Facultad de Ciencias, Universidad Católica del Norte, Antofagasta 1270709, Chile; miguel.cabrera@ucn.cl; 3 EQTrend, Santiago 8320167, Chile; pgonzalez@gmail.com; 4 Subdepartamento de Registro Sanitario de Productos Farmacéuticos Bioequivalentes, Agencia Nacional de Medicamentos, Instituto de Salud Pública, Santiago 7780050, Chile; aaceituno@ispch.cl; 5 Facultad de Farmacia, Escuela de Química y Farmacia, Universidad de Valparaíso, Valparaíso 2360102, Chile

**Keywords:** drug development, models, ADME, BCS, organ-on-a-chip, organoids, machine learning, PBBM, PBPK

## Abstract

**Background/Objectives**: The rapid expansion of New Approach Methodologies (NAMs) is transforming oral biopharmaceutics by offering mechanistically rich, human-relevant tools that can reduce reliance on animal testing while improving translational confidence. Regulatory agencies, including the Food and Drug Administration (FDA) and the European Medicines Agency (EMA), are increasingly open to NAM-generated evidence, provided that methods are fit-for-purpose and scientifically justified. This review synthesizes current advances and evaluates how NAMs can be integrated across drug-development stages to enhance the prediction of oral absorption, formulation performance, and regulatory decision-making. **Methods**: A comprehensive literature review was conducted across classical and emerging methodologies, including in vitro permeability and solubility models, organoids, organ-on-a-chip (OoC) systems, machine learning frameworks, and mechanistic approaches such as the physiologically based pharmacokinetic (PBPK) and biopharmaceutics (PBBM) models. Emphasis was placed on physiological relevance, predictive performance, validation status, and regulatory applicability. **Results**: Classical tools remain essential for the Biopharmaceutics Classification System (BCS)-based biowaivers and risk-based assessments, yet they often lack physiological fidelity. NAMs provide enhanced representation of intestinal architecture, hydrodynamics, transporter activity, and metabolism. Organoids and microphysiological systems generate high-quality permeability and metabolic data, while computational NAMs enable scalable prediction of ADME properties and formulation behavior. When integrated into PBPK/PBBM models, these methods have great potential in predicting in vivo performance in humans. Evidence demonstrates that NAMs can refine, reduce, and, in specific contexts, replace animal studies without compromising scientific rigor. **Conclusions**: NAMs complement, rather than displace, classical biopharmaceutic tools, enabling a more mechanistic, human-centered, and ethically responsible framework for drug development. Their effective implementation will depend on continued validation, standardization, and regulatory harmonization as the field transitions toward fully NAM-supported biopharmaceutical assessment.

## 1. Introduction

The landscape of oral drug development is being reshaped by two converging forces: the need for human-relevant evidence to reduce late-stage attrition and a regulatory shift that encourages scientifically validated alternatives to animal testing. In biopharmaceutics, this means moving beyond descriptive assays toward mechanistic approaches that connect formulation, physiology, and exposure—while aligning with global 3R (Replacement, Reduction, Refinement) commitments [1]. Recent communications from the U.S. Food and Drug Administration (FDA) and European Medicines Agency (EMA) have opened a wider “regulatory window” for New Approach Methodologies (NAMs)—from organoids and organ-on-a-chip to computational tools—provided that they are reliable, fit for context, and demonstrably informative for decisions [2,3]. This moment invites a reassessment of the classical toolkit and a clear articulation of where NAMs can (and cannot) replace or augment existing evidence in support of development and regulatory filings.

Biopharmaceutics is the discipline that studies how the physicochemical properties of a drug, the dosage form, and the route of administration affect the drug’s disposition, which encompasses absorption, distribution, metabolism, and excretion (ADME) [4]. The Biopharmaceutics Classification System (BCS) framework can be used to study such an interaction through the solubility, permeability, and dissolution characterization. Advancements in this discipline have been critical to reduce unnecessary in vivo work via BCS-based biowaivers when strict criteria are met [5]. Global guidance has converged: The International Council for Harmonisation (ICH) M9 harmonizes the scientific and regulatory expectations for BCS classification and biowaivers [6,7]. These anchors ensure that, when fundamentals are satisfied, in vitro data can credibly stand in for in vivo bioequivalence, shortening timelines without lowering standards.

The introduction of the BCS framework to drug development pipelines of more challenging pharmaceuticals with tighter product specifications has resulted in a need of reshaping biopharmaceutic tools to become more mechanistic and predictive of the in vivo situation. Physiologically Based Pharmacokinetic (PBPK) models, and more recently Biopharmaceutics models (PBBMs), integrate formulation inputs (e.g., dissolution/release, precipitation risk) with gastrointestinal (GI) physiology and systemic disposition to simulate plasma/tissue profiles [8,9]. Industry and regulators now routinely discuss using these models to probe sensitivity to dissolution changes, define clinically meaningful dissolution “safe spaces,” and run virtual bioequivalence studies, among many others.

Animal studies have long underpinned in vivo preclinical studies, but their translational limitations for oral absorption (i.e., differences in GI fluid content composition, transporters, absorption mechanisms, luminal hydrodynamics, and metabolism) have been well recognized [10]. At the same time, the ethical mandate to Replace, Reduce, and Refine (3Rs) animal use has become a formal regulatory priority. In the European Union, the EMA explicitly supports 3R-compliant NAMs and offers structured pathways—Innovation Task Force briefings and Scientific Advice—to discuss context of use, fitness for purpose, and evidentiary standards for acceptance [11]. The goal is not a lowered bar but better science: if a human-relevant method is validated, reliable, and appropriate for the decision, it should inform (or replace) animal testing.

In the United States, the FDA Modernization Act 2.0 (29 December 2022) removed the statutory requirement that animal studies be the sole path to first-in-human authorization, explicitly allowing validated alternatives—cell-based assays, computational models—when scientifically justified [2]. In fact, the FDA has recently issued a draft guidance on “General Considerations for the Use of New Approach Methodologies in Drug Development” [12]. Building on this legal foundation, the FDA has announced a plan to phase out certain animal testing requirements, beginning with monoclonal antibodies and selected drugs, and has encouraged inclusion of NAM data in IND submissions as part of a phased roadmap. In parallel, the agency’s public resources now outline streamlined nonclinical contexts where NAMs are acceptable and articulate validation and reliability expectations [12]; the rationale behind these approaches and the level of evidence expected for regulatory use. Even though these steps expand the “regulatory window” for NAMs while preserving rigorous decision-grade standards, the usefulness of NAMs (and their integration) for biopharmaceutic assessments has been only recently explored.

The aim of this review is to provide a coherent, practice-oriented narrative on the role of NAMs in biopharmaceutic assessment throughout drug development and how these methodologies may be used to support development decisions and regulatory applications. We first revisit the classical models of permeability, solubility, and dissolution used for BCS characterization. We then examine the emerging in vitro (i.e., organoids and organ-on-a-chip) and in silico systems (i.e., machine/deep learning, PBPK, and PBBM) and their potential to better predict drug (product) biopharmaceutics. Finally, we outline regulatory adoption pathways and pragmatic integration strategies that align with the 3Rs and with current FDA/EMA positions, emphasizing contexts where NAMs can credibly replace, reduce, or refine animal studies while improving human translatability. While the concepts and frameworks discussed herein are broadly relevant to oral biopharmaceutics, this review primarily focuses on small-molecule drugs administered via the oral route, for which permeability, solubility, dissolution, and intestinal metabolism are dominant determinants of systemic exposure and regulatory decision-making. The discussion is therefore centered on human intestinal absorption of low-molecular-weight compounds, particularly within the context of the Biopharmaceutics Classification System (BCS), permeability-based risk assessment, and formulation optimization.

Although selected examples from peptides, enabling formulations, or complex delivery approaches are cited where mechanistically informative, this review does not aim to comprehensively cover biologics, locally acting gastrointestinal products, or non-oral routes of administration. Within this defined scope, New Approach Methodologies (NAMs) are evaluated based on their ability to generate quantitative, translatable parameters suitable for integration into physiologically based pharmacokinetic (PBPK) and biopharmaceutics models (PBBM) and for their potential regulatory applicability in small-molecule drug development.

## 2. Classical Models in Oral Biopharmaceutics

### 2.1. Permeability Determination in Humans and Classification

The Biopharmaceutics Classification System (BCS) was proposed by Amidon et al. [13] and subsequently adopted by different regulatory agencies worldwide (ICH M9, FDA, and EMA) [5,6]. The bases of the BCS were derived from the correlation between in-human effective permeability (P_eff_) and drug fraction absorbed. The efforts in developing such a dataset were invaluable, mainly because of the high complexity associated with intestinal perfusion techniques used to determine drug P_eff_ in humans. Although different techniques can be used to determine intestinal permeability, most intestinal P_eff_ values reported in the literature have been determined with the Loc-I-Gut method [14]. This method was developed in the late 80s at the University of Uppsala [15], and it consists of intubating subjects with a two-balloon system. Once balloons are located in the upper small intestine, they are inflated, isolating an intestinal segment of 10 cm [16]. The drug is subsequently perfused, and the disappearance of luminal concentrations can be used to calculate P_eff_, as per Equation (1):

P_eff_ = Q_in_ × [(C_in_ − C_out_)/(C_out_ × A)], (1)

where Q_in_ is the perfusion flow rate, C_in_ and C_out_ are input and output concentrations across the isolated segment, and A is the surface area assuming a cylindrical geometry. The Loc-I-Gut method is considered the gold standard method for determining drug permeability in humans. However, intubating subjects carries many concerns, including ethical aspects, experimental times, and costs. Consequently, few data have been published with this technique in recent years [17]. In fact, several surrogate techniques for determining drug permeability have been implemented for drug development, involving in vivo assays in pre-clinical models, in vitro cellular and non-cellular models, and in silico techniques. The reader is referred to the recently published article by Koziolek et al., who nicely reviewed the state of the art of these methods [17,18,19].

In order to classify drug permeability as per the BCS, the drug permeability coefficient (e.g., P_eff_) should be compared to the P_eff_ of a known permeability marker for which its fraction absorbed (Fa) is 85%. Although metoprolol has been the gold standard marker, that comparison is still somewhat conservative, as it exhibits an Fa of 90%. Alternatively, drug permeability classification can be obtained from an absolute bioavailability trial, where a bioavailability (F) higher than 85% suggests the drug was highly permeable. Although less invasive, this method is limited to drugs with negligible first-pass effect and/or bile excretion.

### 2.2. Animal Pre-Clinical and Animal-Derived Ex Vivo Models

In order to decrease human trials, animal pre-clinical models are used at early stages of drug development to select compounds with favorable pharmacokinetic (PK) and biopharmaceutical attributes. Animal models are chosen to provide results that can be extrapolated to humans. Accordingly, rodents and canines are among the most used models for drug absorption assessment. Unlike standard static in vitro permeability assays (see below), in vivo models can better resemble the complexity of the GI tract, including dynamics and gradients in fluid composition.

On the one hand, the dog model is widely used not only because it shares some similarities with human physiology/anatomy but also because it can be administered with human-size formulations, such as oral pharmaceutical solid dosage forms [20]. For instance, the usefulness of the beagle as a model for pediatric administration was recently supported using two highly permeable model drugs [21]. Dog bioavailability has been previously compared to humans, achieving an acceptable correlation (r^2^ = 0.8). However, while that correlation was stronger for high-permeability compounds, dogs overall tend to overestimate human F for low-permeability drugs [22]. This is because the canine intestine tends to be leakier with respect to drug permeation than the human gut. Possible explanations may include the much more alkaline stomach pH in dogs (pH = 6.1–7.6), shorter transit times and transporter abundances, among others [20,23]. For instance, Matsumura et al. showed that human Fa predictions from dog data are not appropriate for acidic compounds due to discrepancies in gastrointestinal pH values affecting drug solubility [24]. The mismatch between human and dog bioavailability may also relate to more abundant paracellular pores in dogs, having a radius that is also estimated to be larger than in humans [25]. An attempt to identify the type of drugs an extrapolation from dogs to humans is acceptable was conducted. The authors found that drug molecular charges (acid, base, etc.), molecular weight, and primary elimination mechanism (e.g., hepatic vs. renal) were important factors to understand discrepancies between human and dog bioavailability [26]. Considering these differences, the closer resemblances of NAMs to animal and human physiology (e.g., higher luminal pH in dogs or faster metabolic rates in mice) offer a possibility for studying neutral, acidic, and basic drug molecules, thus enhancing the accuracy in preclinical to human extrapolation.

On the other hand, the rat model holds a prevalent role in PK studies due to their manageable nature, ease of dosing, and the opportunity for sequential blood sampling from a single animal. However, a rat’s compact size does not allow the administration of human-size oral solid dosage forms, and hence, rat models are usually administered with oral solutions and/or suspensions via oral gavage. Even though rat and human physiologies share some common features, like gastric emptying times, there are some marked differences, including a slightly more alkaline pH in the stomach (pH 4–7) and higher intestinal bile salt concentrations (33–61 mM) [23]. While these factors are mainly expected to affect drug solubility, rat models are most usually employed to study drug permeability. In fact, the in situ single-pass intestinal perfusion (SPIP) and Doluisio’s (closed-loop) perfusion techniques have shown good correlations with human P_eff_, although rat P_eff_ values tended to be lower [27,28]. These techniques were also used to explore regional drug absorption [29,30] and the effect of excipients [31,32,33,34]. One of those studies compared the effect of permeability enhancers on different model drugs after using either SPIP or intestinal bolus administration. The results indicated the SPIP technique was more sensitive to the permeability-enhancing effect than the intestinal bolus [34], and hence, it may overestimate the effect of excipients in humans [35]. The performance of the intestinal bolus technique was also compared between dogs and rats. P_eff_ values in dogs were not only higher than in rats but also overall less affected by permeability enhancers [34]. Last but not least, it is known that rat models perform better in predicting P_eff_ than human oral bioavailability, given the large differences in enzyme levels and expression in the gastrointestinal tract [23,36].

Excised rat intestinal tissue has also been mounted on the Ussing Chamber to perform ex vivo assessment of intestinal drug permeability. This is a two-compartment chamber where the donor and the receiver are separated by the animal tissue [37]. Mounting rat tissue on the Ussing Chamber improves the resemblance of the intestinal mucosa, since both subepithelial tissue and villi expansion are present in excised tissue [14]. In fact, rat jejunum apparent permeability (P_app_) values for several model compounds correlated well against human P_eff_ data [38,39]. Intestinal tissue from other species, for instance, pigs, has also been mounted on similar devices to study drug permeability [40,41].

Overall, differences between human and animal permeability models have been revised by other authors, and the reader is referred to those articles for further information [14,23].

### 2.3. In Vitro Models: Permeability, Solubility, and Dissolution/Release

Regulatory guidelines include in vitro permeability testing as a surrogate method for permeability classification, provided demonstration of method suitability [6]. In vitro methods typically consist of seeding an epithelial cell monolayer (i.e., Caco-2 cells [42] or Madin–Darby Canine Kidney [43], MDCK cells) onto a porous membrane, thus separating donor from receiver compartment. Prior to the experiment, the donor compartment is filled with drug solution at the donor concentration (C_d_), and samples are collected from the receiver compartment. When sink conditions are maintained, drug appearance in the receiver compartment can be used to calculate flux (J [mass/time × area]), and therefore, apparent permeability (P_app_) can be calculated from Equation (2).

(2) $$J=P_{app}\times C_{d}$$

This experimental setup is usually mounted on Transwells^®^ (Corning, NY, USA), which are polycarbonate inserts compatible with standard plastic wells. Throughout the study, the donor solution is kept in the donor compartment; thus, the system is static in nature. Even though this assay does not resemble in vivo dynamics (where the drug is transported from the proximal to distal gastrointestinal tract by convective fluxes), they have shown strong correlations with in vivo fraction absorbed (Fa) and human permeability [43]. As a consequence, the static in vitro permeability assay mounted with a suitable epithelial cell model has been widely accepted by regulatory agencies and the pharmaceutical industry.

The importance of the BCS relates to the scientifically sound framework it provides for waiving in vivo bioequivalence testing. Accordingly, a drug must be classified not only in terms of permeability but also in terms of solubility. Solubility is defined as the maximum amount of drug that can be dissolved in a given solvent. Gastrointestinal fluids are aqueous-based media for which their pH varies from acidic (pH 1–2) to neutral (pH 6–8) from the proximal to distal tract [44]. Regulatory guidelines recommend demonstrating drug solubility in simplified aqueous media, such as hydrochloric acid, acetate, and phosphate at pH 1.2, 4.5, and 6.8, respectively [45]. However, fasted and fed state simulated intestinal fluids (FaSSIFs and FeSSIFs), as well as fasted and fed state simulated gastric fluids (FSSGFs), have emerged as a more biologically relevant (biorelevant) alternative, mimicking not only the fluid’s pH but also osmolality, ionic strength, and bile salts/phospholipid contents [46,47]. These media have been validated against human intestinal fluid (HIF) obtained from intestinal aspirates [48]. Although FaSSIF and FeSSIF can be considered more biorelevant than regulatory media, concerns have emerged about the role of intestinal buffering capacity on solubility assessment. For instance, Claussen et al. recently showed that enhanced bulk buffer capacity in the intestinal lumen, due to constant bicarbonate secretion, may lead to higher saturation solubility values for ibuprofen, a model-weak acidic drug, compared to phosphate [49]. The results suggest that even solubility measurements in non-sparged HIF may underestimate true in vivo solubility, which may have consequences on BCS solubility classification for some active pharmaceutical ingredients (APIs). This has caused a mismatch between dissolution and solubility, consistent with findings presented by Claussen, Holzem et al., where dissolution was studied under large dose-to-volume ratio conditions [49,50]. Therefore, more research is needed to further design more biorelevant and biologically predictive (biopredictive) methods for intestinal solubility assessment.

In the late stage of development, formulation performance is usually tested in dissolution testing using compendial apparatuses. This allows not only the testing of formulation performance but also the definition of the dissolution method and quality control (QC) specifications. While BCS-based biowaiver methods recommended the use of compendial media at biorelevant pH values (see above), FaSSIF, FeSSIF, and even bicarbonate-based methods have been used to further increase biorelevance in certain cases [51,52]. Although very useful in assessing drug dissolution, the pharmacopeial assay is not sensitive to the interplay between dissolution and permeation, as it actually occurs throughout the small intestine during the drug absorption window. In this regard, dissolution/permeation (D/P) systems have been developed to better capture the dynamic nature of drug oral absorption in vivo, where dissolution/release of the drug and permeation across the intestinal membrane take place simultaneously. This dynamic is crucial to study oral absorption of BCS II drugs or dissolution-limited products since permeation can drive dissolution [53,54]. The basic setup of a D/P consists of a dissolution compartment (donor), a membrane, and a receiver chamber. Since the seminal work of Kataoka et al. [55], several different modifications have been published. Typically, Caco-2 or MDCK cell monolayers have been used as a membrane, but excised intestinal tissue and artificial membranes have also been explored [56,57,58]. Donor solutions, including aqueous buffers and biorelevant media (FaSSIF/FeSSIF), have been used with and without gastric fluid pre-treatment [59,60]. Bovine serum albumin (BSA) or surfactants have been added to the receiver chamber to favor sink conditions [61]. Lately, some proprietary setups have adapted the receiver chamber into vessels compatible with standard dissolution apparatus [62,63]. Even though adaptations of standard methods have resulted in more biorelevant tools to study drug product performance, New Approach Methodologies (NAMs) may be leveraged to increase the predictive potential of in vitro and computational methods. The following section reviews the advances of NAMs applied to assess drug (product) biopharmaceutics.

## 3. Biopharmaceutic Applications of New Approach Methodologies

### 3.1. In Vitro Systems

#### 3.1.1. Physiological Fidelity Through Organoids

From a historical perspective, the transition toward increasingly physiological models should be viewed as incremental rather than disruptive. Classical epithelial monolayer systems—most notably Caco-2 cells and their variants—have long served as the reference standard for intestinal permeability assessment, owing to their robustness, reproducibility, and demonstrated correlation with the human fraction absorbed [64]. Extensions of this approach, such as Caco-2/HT29-MTX co-culture models, were introduced to incorporate mucus-secreting goblet-like cells, thereby enabling improved evaluation of transmembrane transport for compounds sensitive to the unstirred water layer or mucus barrier [65].

While these co-culture systems represent an important refinement over monocultures, they remain fundamentally static and lack important physiological features, including dynamic flow, regional differentiation, and native transporter abundance [66]. This limitation often results in inaccurate predictions for low-permeability drugs and transporter-mediated absorption. The need for human-relevant, mechanistic platforms has driven the development of NAMs, which combine advanced in vitro systems, microfluidics, and computational modeling.

Organoids are three-dimensional, self-organizing structures derived from stem cells or patient biopsies, representing a paradigm shift in preclinical modeling. By recapitulating the complexity of native tissues, organoids provide unprecedented physiological fidelity, enabling mechanistic studies of absorption, metabolism, and disease pathophysiology. Intestinal organoids reproduce crypt-villus architecture and express clinically relevant transporters (MDR1, BCRP, and PEPT1) and enzymes (CYP3A4), which are absent or poorly represented in conventional monolayers [67]. A key milestone was the demonstration that murine and human intestinal organoids retain functional nutrient and drug transporters, enabling studies of uptake, sensing, and secretion within an epithelial context close to in vivo physiology. The first attempts to use intestinal organoids for transport studies emerged in 2015 by Zietek et al. [68,69]; however, this phase also exposed a structural paradox: classical intestinal organoids formed closed cystic spheres, with the apical surface facing an enclosed lumen. While suitable for microinjection-based studies, this geometry was fundamentally incompatible with standard apical-to-basolateral permeability assays [70]. The major breakthrough for drug absorption research came with the development of organoid-derived epithelial monolayers. By dissociating 3D organoids and re-seeding them onto Transwell membranes, researchers generated polarized, accessible epithelia that preserved donor-specific gene expression, lineage diversity, and physiologically relevant tight junction architecture [66]. This innovation effectively bridged the gap between organoid biology and pharmacokinetic methodology. Studies during this period showed that organoid-derived monolayers display permeability values, TEER ranges, and transporter activity that more closely resemble human intestinal tissue than Caco-2 cells [66].

**Figure 1:**
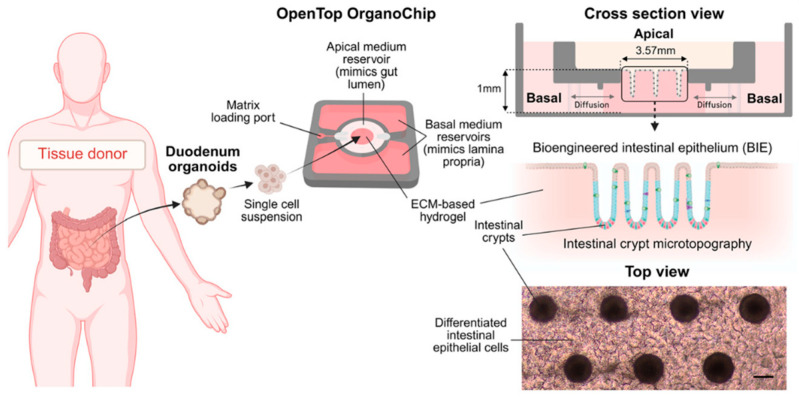
The OpenTop OrganoChip approach. Taken from [72].

In the last five years, intestinal organoids have become explicitly embedded in drug development workflows, rather than experimental or research tools. Multiple groups demonstrated that organoid-derived models can reproduce passive permeability, active transport, and intestinal metabolism simultaneously, enabling joint estimation of Papp, transporter effects and gut-wall extraction. Current advanced systems combine, for example, microfluidic flow (organoid-on-chip platforms) and PBPK and mechanistic ADME modeling [71,72,73,74]. This integration allows permeability data from organoids to be translated into clinically relevant Fa estimates, often with higher correlation with human data than traditional assays. For example, human duodenal organoids were combined with microfluidics to develop the “OpenTop OrganoChip” (Figure 1) [71,72]. This model achieved CYP3A4 induction, transporter activity, and crypt-villus organization—predictive of in vivo performance—with the potential not only to correctly classify APIs according to the BCS but also to assess drug metabolism and pharmacokinetics (DMPK) performance [71].

Organoids have rapidly evolved from static three-dimensional cultures into dynamic, data-rich platforms that integrate biology, engineering, and computational science. One of the most transformative advances has been the application of CRISPR-Cas9 gene editing, which enables precise modeling of genetic disorders and facilitates target validation in drug discovery. This capability allows researchers to generate organoids that replicate disease-specific phenotypes, creating highly relevant systems for therapeutic screening [75]. Equally significant is the incorporation of multi-omics technologies, including transcriptomics, proteomics, and metabolomics, which provide a holistic view of drug responses at multiple biological levels [76]. These approaches uncover molecular signatures of efficacy and toxicity that traditional models often miss, positioning organoids as powerful tools for mechanistic studies. This can be attributed to the preservation of segment-specific transporter and enzyme expression in human intestinal organoids (e.g., PEPT1, OATP, P-gp, BCRP, CYP3A4), allowing permeability and first-pass intestinal metabolism to be evaluated together rather than in isolation. This contrasts with Caco-2 models, which often misrepresent transporter abundance and exhibit non-physiological tight junctions, leading to biased estimates of the fraction absorbed [77,78].

To address challenges of reproducibility and scalability, internal simplification, such as chemically defined media and engineered scaffolds, is combined with external enhancements like microfluidics, bioprinting, and AI-driven analytics [79]. This integrated strategy improves consistency across laboratories while enabling real-time monitoring and predictive modeling. Advances in bioprinting and automation have further expanded organoid applications by enabling high-throughput screening and even single-organoid resolution assays [80]. These technologies reduce variability, accelerate experimental timelines, and support large-scale drug testing campaigns. Complementing these developments, organoid-on-a-chip systems have emerged as hybrid platforms that merge the architectural complexity of organoids with the precise environmental control and perfusion capabilities of organ-on-a-chip (OoC) devices. This convergence enhances physiological relevance, supports multi-organ interactions, and facilitates integration with computational models, marking a critical step toward predictive, translational pharmacology.

Intestinal organoids remain central for studying absorption and transporter-mediated processes under physiologically relevant conditions, but their utility extends far beyond the gut. Liver organoids are increasingly used to predict drug-induced liver injury, offering superior accuracy compared to animal models [81]. Similarly, neural organoids provide unprecedented opportunities to model neurotoxicity and neurodegenerative diseases, supporting the discovery of therapies for complex central nervous system disorders [82]. Furthermore, in oncology, patient-derived organoids (PDOs) have revolutionized precision medicine by enabling individualized predictions of chemotherapy and immunotherapy responses. These models preserve the genetic and phenotypic heterogeneity of tumors, allowing clinicians to tailor treatments based on real-time functional data [83]. Beyond single-organ systems, researchers are now developing multi-organ platforms that integrate gut, liver, and kidney organoids to capture holistic ADME processes [84,85]. Efforts to incorporate vascular networks and immune components are addressing critical gaps in modeling systemic drug effects and immunomodulation, paving the way for applications in immunotherapy development and personalized dosing strategies [86]. Importantly, these studies also highlighted a sobering reality: increased biological realism does not automatically yield better predictions unless coupled with appropriate modeling frameworks [87].

#### 3.1.2. Dynamic Microenvironments with Organ-on-a-Chip

While organoids provide cellular complexity, organ-on-a-chip systems introduce dynamic flow and mechanical cues that static models cannot replicate. These platforms recreate physiologically relevant hydrodynamics, enabling more accurate predictions of drug absorption and metabolism. The concept was pioneered by Kim et al. in 2012, who developed the first gut-on-a-chip featuring two microfluidic channels separated by a porous membrane [88]. By applying laminar flow and cyclic strain, the system promoted villus formation, mucus secretion, and enhanced barrier integrity compared to conventional static cultures. This approach was extended by incorporating a porous nitrocellulose membrane and constant flow to study intestinal metabolism of drugs such as verapamil and ifosfamide [89].

**Figure 2:**
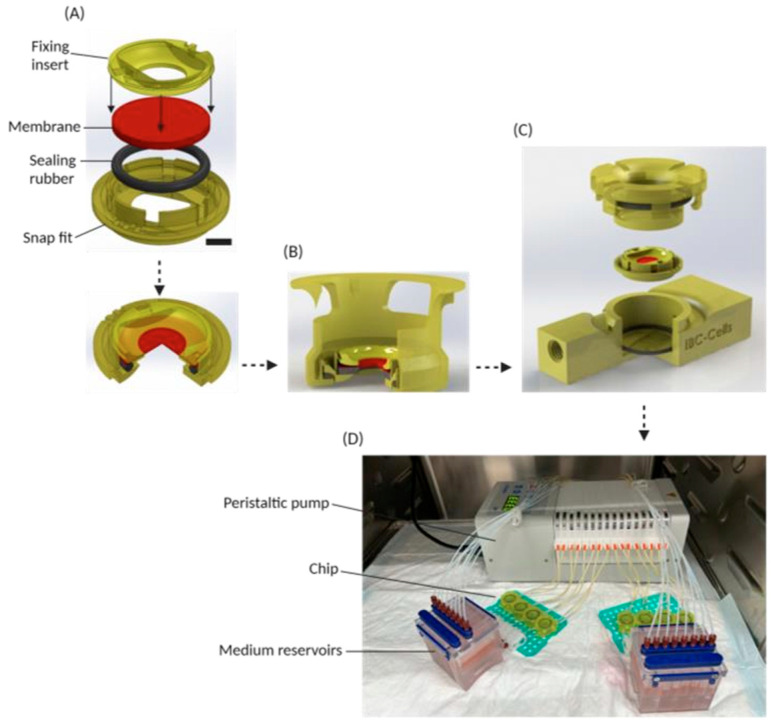
Cells-on-chip system described by Keuper-Navis et al. (**A**) In-house developed 3D-printed disc with porous membrane and rubber ring for cell monolayer culture. Scale bar equals 2 mm; (**B**) Disc in a carrier for cell seeding and monolayer formation (static culture); (**C**) The disc with a cell monolayer can be transferred to a chip with dual flow; (**D**) Complete cell-on-chip system set-up, with eight chips connected to separate apical and basolateral medium reservoirs, with a peristaltic pump to create laminar flow, placed inside an incubator. Taken from [91].

Recent innovations have focused on modularity and physiological relevance. Eslami Amirabadi et al. introduced a modular chip design that is able to fix tissue explants between two microchannels, also called the “Intestinal explant barrier chip (IEBC)” system [90]. Using human and porcine colon tissue, the IEBC system was demonstrated to be a suitable model by correlating drug permeability of seven FDA-approved model compounds to their fraction absorbed in humans. Later on, Keuper et al. adapted this modular chip design (cells-on-chip system), allowing the use of a static culture of Caco-2 and enteroid monolayers before transitioning to dynamic conditions (Figure 2) [91]. Their work revealed that laminar flow significantly influences permeability: Atenolol and metformin exhibited higher P_app_ under flow, while antipyrine permeability decreased compared to static conditions. While in vitro permeability coefficients between low and high permeability markers typically differ by orders of magnitude, the fraction absorbed of antipyrine was only around 2-fold higher than that of atenolol. Although the mechanisms have not yet been investigated, to the best of our knowledge, we believe one possible explanation is the interplay between convective flow in the proximal-to-distal direction competing against radial drug aqueous diffusion. Given that Keuper-Navis et al.’s modular chip design was able to shorten such a gap in permeability coefficients, this model has great potential for assessing permeability in a more biorelevant fashion [91].

The commercial chip system developed by Emulate Bio, Inc., has also been used to develop predictive permeability models. The device is made out of polydimethylsiloxane (PDMS) and comprises two microfluidic channels for apical and basolateral layers, as well as a porous PDMS membrane for cell culturing [92]. The gut-on-a-chip (GoC) system consisted of Caco-2 cells mounted onto this device, and it was used to study drug permeability. Method suitability was proven by classifying 19 FDA-listed compounds according to their Fa values. Similar to other organ-on-a-chip devices, this method was able to reduce the large difference between low and high permeability markers, typically exhibited by static cell models. This was demonstrated with the differences between antipyrine and nadolol and high- and low-permeability model compounds, respectively. These drugs showed a 500-fold increase in their P_app_ values when tested in the static inserts, whereas that gap was reduced by 92% when assessed in the GoC system. The same system was later applied to study the effect of the permeability enhancers on drug permeability of oral peptide formulations [93]. While static models foresee the great potential of permeability enhancers, oral peptide products such as Mycapssa^®^ (Chiesi, Parma, Italy) and Rybelsus^®^ (Novo Nordisk, Bagsværd, Denmark) have relied on this type of excipient to display only a slight increase in their oral absorption. Hence, the static insert Caco-2 model was compared to the aforementioned GoC system on the effect of permeability enhancers, sodium caprate, and sucrose monolaurate. As expected, these excipients showed a lower, yet significant, enhancement of P_app_ of model peptide compounds in the GoC compared to the standard Caco-2 system. These findings further support the higher biorelevance of dynamic than static permeability systems.

Building on these foundations, a human duodenum-on-a-chip was recently established as a surrogate for effective human permeability [74]. Using biopsy-derived organoids integrated into this device, this study showed that in vitro P_app_ of the model compounds, lisinopril, metoprolol, and fluconazole, correlated with the P_eff_ measured in humans. The duodenum intestine-on-a-chip was able to show not only good correlations, but it also confirmed the expression of physiologically relevant proteins, such as CYP3A4 and P-gp.

Collectively, these advances underscore a critical insight: flow is not merely a technical feature, but it is a critical biological aspect in determining drug absorption. By modeling hydrodynamics, mechanical forces, and segment-specific physiology, organ-on-a-chip systems provide a more accurate representation of intestinal function than static models.

### 3.2. In Silico Systems

#### 3.2.1. Transitioning from Classical QSPR to Deep Learning Models

**Figure 3:**
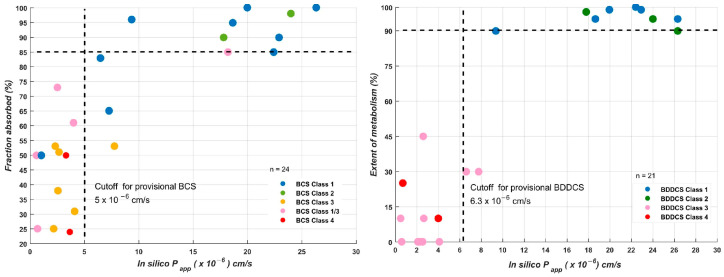
Relationship between in silico apparent permeability coefficients and fraction absorbed (**left** panel) or extent of metabolism (**right** panel) for 32 model drugs of the ICH list, classified according to BCS or BDDCS classification systems, respectively. Dashed lines represent the cutoff values for either BCS (Fraction absorbed 85%) or BDDCS (Extent of metabolism 90%). Taken from [95].

Computational methods have evolved from simple linear correlations used to predict thermodynamic solubility (logS), lipophilicity (logP/logD), and ionization constants (pKa), which remain essential for provisional BCS classifications, to sophisticated machine learning (ML) architectures capable of forecasting complex ADME endpoints with high physiological relevance (Figure 3) [94,95]. Current state-of-the-art models, particularly those employing deep learning (DL) and graph neural networks (GNNs), are capable of forecasting oral bioavailability (F) [96], in vitro apparent permeability (P_app_) across Caco-2 monolayers [95], and the percentage of human intestinal absorption (%HIA) [97]. Furthermore, these in silico tools accurately estimate distribution and clearance parameters, including the fraction unbound in plasma (fu) [98], the volume of distribution (Vd) [99], and cytochrome P450-mediated intrinsic clearance (CL_int_) [100]. These in silico NAMs provide an unparalleled scale for screening thousands of candidates in the early stages of drug development, capturing non-linear relationships that traditional QSPR models often fail to identify.

However, it is important to recognize that ML/DL and GNN-based models are inherently dependent on the quality and representativeness of the underlying datasets. Dataset heterogeneity, bias, and extrapolation beyond the training domain are well-recognized challenges but are not unique to data-driven approaches and similarly affect experimental ADME datasets and classical QSPR models. Current best practices address these limitations through rigorous data curation, standardized preprocessing, and validation against independent external datasets, which are now considered essential components of robust model development [101,102]. In addition, the explicit definition of the domain of applicability (DoA), based on chemical similarity and descriptor space coverage, ensures that predictions are restricted to reliable regions of chemical space [103,104]. Complementarily, uncertainty quantification strategies, including ensemble learning approaches, enable the identification of low-confidence predictions and reduce the risk of uncritical extrapolation [105]. Importantly, extrapolation to novel chemical space, particularly for compounds with underrepresented mechanisms, remains a recognized limitation of purely data-driven models. In this context, hybrid strategies integrating ML/DL predictions with mechanistic frameworks, such as physiologically based pharmacokinetic (PBPK) models, have emerged as a robust solution, improving predictive performance while maintaining physiological interpretability [106,107]. This integrative paradigm is increasingly regarded as a best-practice approach to ensure both predictive reliability and biological relevance in modern ADME modeling.

Beyond the application of ML and DL models to predict key thermodynamic parameters for predicting biopharmaceutics, their application has been expanded to formulation design and assessment. For instance, dissolution profiles were predicted with ML models for 377 tablet formulations with different (immediate, sustained, and controlled) release mechanisms [108]. Though useful for predicting dissolution kinetics, the strength of ML models lies in formulation design. This was nicely demonstrated by Garamani et al., who used ML algorithms to guide the rational design of ibuprofen tablets. In this approach, the ML model provided insights on the role of critical manufacturing attributes (CMAs) and critical process parameters (CPPs) on ibuprofen tablets’ PK [108].

Further studies have shown the potential of artificial neural network (ANN) models. For instance, an ANN model was recently used to develop a metronidazole sustained-release formulation by optimizing critical manufacturing attributes (CMAs) and critical process parameters (CPP) to modulate release kinetics [109]. ANN architectures have also been constructed to develop data-driven dissolution models, which were compared to mechanistic models. Although the ANN model outperformed the more mechanistic approach for the specific case of immediate release tablets, the authors concluded that hybrid approaches may be ideal as they could “exploit the explainability and extrapolation” strengths of each modelling approach [110]. In fact, a mechanistic model including an “accelerating factor” showed better performance than ANNs for the case of sustained-release formulations [111]. Hence, the evidence supports future directions for developing hybrid models, such as physics-informed neural networks (PINNs) for predicting tablet dissolution performance.

#### 3.2.2. Mechanisms Underlying Drug Absorption: Physiologically Based Pharmacokinetic Models (PBPK)

Nowadays, PBPK and PBBM models are perhaps the most explored NAM tools in biopharmaceutic assessments. The underlying idea of PBPK models was first developed by Tosten Teorell in 1937 [112], who developed and solved differential equations for drug distribution and elimination processes, including permeation coefficients as the driving force for drug-to-tissue mass transfer. Nowadays, PBPK are mechanistic models with the potential to simulate plasma and tissue concentration versus time profiles from physicochemical and biological inputs. The most widely used PBPK approaches are mounted on commercial (and even some free-access) platforms, such as Simcyp^®^ (Certara, Inc. Radnor, PA, USA), GastroPlus^®^ (Simulations Plus, Inc. Landcaster, CA, USA), and PK-Sim^®^ (Open Systems Pharmacology). Nonetheless, PBPK models have also been coded on other wider platforms such as MATLAB^®^ (MathWorks, Natick, MA, USA) or Berkley Madonna^®^ (Albany, CA, USA). Models used to simulate tissue drug concentrations include the models of Poulin–Theil, Rodgers–Rowland, and Lukacova, among others [113,114,115,116]. While those models handle post-absorptive metabolism/distribution/elimination, physiological models of the GI transit have been developed to simulate oral drug absorption. Said models compartmentalize the gastrointestinal tract according to segments with different physicochemical features and assign kinetic coefficients to drive mass transfer between compartments.

Drug absorption of small molecules takes place via one or a combination of mechanisms, including passive drug diffusion, paracellular permeation, and transporter-mediated active transport. Accordingly, PBPK models have been used to understand drug absorption mechanisms. The “MechP_eff_” model was developed to account for passive and paracellular, as well as cases where drug absorption is limited by the unstirred water layer [117,118]. The main input parameter in this model is transcellular permeation, which can be obtained from in vitro P_app_ determinations in Caco-2 cells. Alternatively, Caco-2 vs. LogP or vs. PAMPA correlations may be used to estimate in vitro cell permeability. Although this model successfully predicted in vivo P_eff_, its reliability on Caco-2 cells input data may cause inaccuracies in predictions [117]. This may be caused not only by the static nature of the standard Caco-2 permeability method (see above) but also by the high inter-laboratory variability reported with outcomes when using that cell line [119]. Alternatively, the Advanced Compartmental Absorption and Transit (ACAT) model [120] has included the models of Adson and He to account for paracellular permeability [121,122]. This model was used to elucidate the role of paracellular permeability on the absorption of minoxidil and acyclovir, among other drugs [123,124]. Furthermore, PBPK models have supported the hypothetical mechanism of lysosomal trapping in delaying drug absorption of highly basic lipophilic drugs [125,126,127]. These approaches included the potential role of lysosomal trapping by modifying the fraction unbound in the enterocyte matching simulations with observed plasma curves. Considering the in vivo complexity of this process, further research is needed to validate this approach.

Another advantage of mechanistic PBPK models is their potential for investigating the role of transporter-mediated active transport on drug absorption. While in vitro clearance has been used (and even criticized) to extrapolate in vivo clearance [128] through the application of a universal scaling factor [129,130], this is much more difficult for transporters. The reasons include polarized transporter expression, the role of driving force co-factors in vitro vs. in vivo, and the gap in evidence for quantitative expression of transporters. Though recent advances in transporter abundance have contributed [131,132,133,134], the latter point remains a challenge because of potential non-linearities between transporter abundance and activity [135,136]. This issue may be even more challenging if models of specific populations or patients were attempted [137,138,139]. In spite of those limitations, some cases of success have been reported on the use of PBPK to study the contribution of transporter-mediated active transport to drug absorption [116,140,141,142,143].

Other recent applications of PBPK to study drug absorption include the interplay between transit times and transporter kinetics [144,145,146], the development of advanced models to predict regional permeability throughout the GI tract [147,148], and the effect of acid-reducing agents (ARAs) on drug absorption [149,150].

As mentioned, permeability input in these models usually comes from in vitro studies in standard static models. This results in large prediction errors for low-permeability drugs, such that permeability optimization against in vivo data has become a common strategy when developing PBPK models. Therefore, integration of other NAMs with PBPK models has become an attractive solution. The duodenum-on-a-chip recently developed was integrated into a PBPK platform to simulate systemic exposure, thus replacing the static Caco-2 cell permeability input by a more physiological one [74].

**Figure 4:**
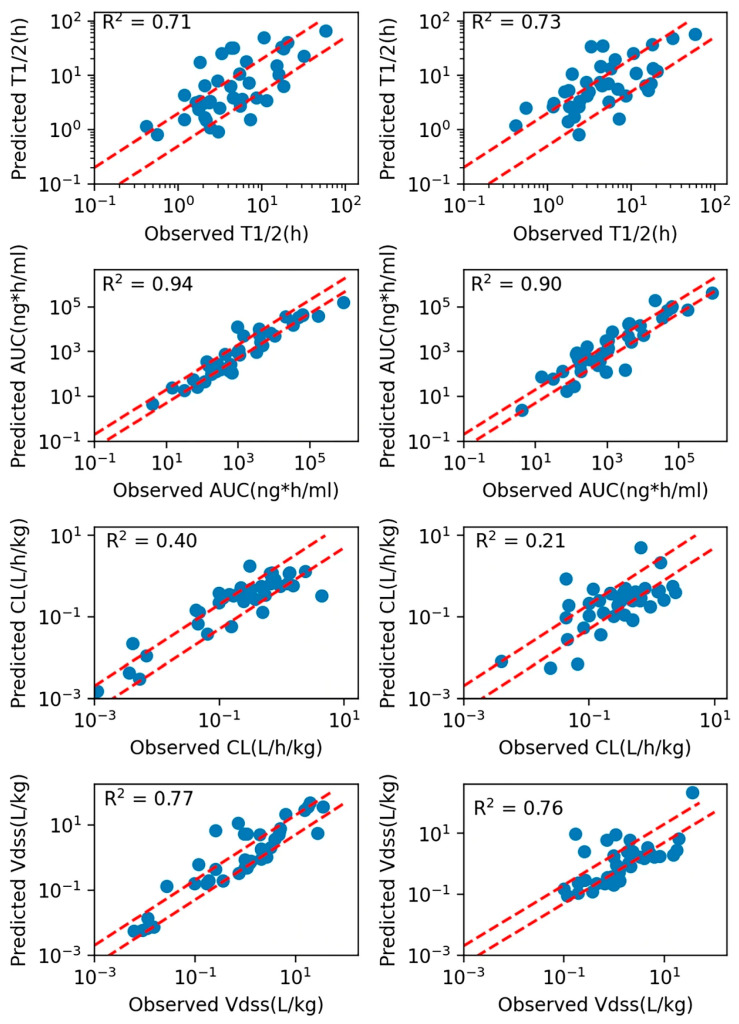
Comparison between predictions and observations for PK parameters after IV dosing in humans using ML inputs (**left**) and in vitro inputs (**right**). Two red dashed lines represent ± two-fold errors. R^2^ values were the Pearson correlation coefficient values. Taken from [153].

A similar workflow can be applied, where QSPR and ML models serve as a critical “entry point,” providing essential input parameters such as fraction unbound (fu), volume of distribution (Vd), or intrinsic clearance (CLint) when experimental data from biological NAMs are not yet available [151]. This synergy between in silico predictions and PBPK modeling allows for early-stage pharmacokinetic projections that guide the selection of candidates for more resource-intensive organ-on-a-chip studies, transforming NAMs into a continuous translational pipeline from bits to cells [152]. For example, Li et al. combined an ML model for plasma protein fu and Caco-2 permeability parameters with a PBPK to predict exposure of different small molecules [153]. Interestingly, the model produced highly accurate predictions, hence demonstrating the strength of integrating different NAMs to guide drug development (Figure 4).

#### 3.2.3. Modeling the Interplay Between Formulation and Absorption: PhysiologicallyBased Biopharmaceutics Models (PBBMs)

Understanding drug absorption mechanisms is key to guiding the rational formulation strategy. In this regard, PBPK modeling has been coupled with biopharmaceutic assessment of formulations (e.g., dissolution kinetics, release mechanisms, effect of excipients, among others) to understand the interplay between formulation physicochemical features and physiological aspects.

In one example, the effect of excipients on drug bioavailability was assessed through sensitivity analyses for several model compounds using a physiologically based model [154]. The authors found that changes in solubility had minimal impact on the simulated bioavailability of BCS I and III compounds. Conversely, the interplay between passive diffusion and transporter activity plays a key role and demands further understanding. For instance, the enhancing effect of Cremophor RH40 on digoxin bioavailability was successfully simulated in the model by either increasing digoxin permeability or reducing P-gp activity [154]. Similarly, the osmotic effect of sugar alcohols, mannitol and sorbitol, was estimated in a validated PBBM model. The findings suggest that sugar alcohol doses of up to 400 mg have a negligible impact on oral bioavailability PK parameters [155]. Although these examples did not integrate in vitro permeability measurements with the PBBM, they showcase the potential of PBBM models in studying excipient effects without in vivo models. Furthermore, outcomes of this type of research may be extremely helpful in making decisions at rather late stages of drug development. The oversensitivity of static models to the effect of excipients is perhaps the main limitation for successfully integrating permeability in vitro observations into PBBM [156,157]. We foresee that future evidence using in vitro NAMs may provide more reliable simulations for studying the effect of excipients on drug absorption. Another limitation is the risk associated with interpretability and parameter identifiability when modeling the effect of excipients through adjusting alternative parameters. Although this strategy may be a surrogate method for the lack of mechanistic models on the effect of excipients, the presence of evidence that supports said parameters, which is actually related to the effect of excipients, is critical. Again, in vitro NAMs find a niche here in improving confidence in the mechanisms underlying the excipient effect.

Currently, the most common implementation of PBBM consists of using dissolution/release profiles as input functions into the PBPK model [8,158]. With this approach, the PBBM can be used to set dissolution specifications, design a biorelevant/biopredictive dissolution method, and build a dissolution safe space, among others. For these applications, it is usual to find middle-out modeling strategies, as was the case with ciprofloxacin [159]. However, Tarumi et al. proposed that the consideration of the unique physicochemical behavior of bicarbonate, the physiological intestinal buffer, on drug dissolution can significantly improve predictions of PBBM when using bottom-up approaches [159]. In fact, PBBM and mass transfer modeling have shown that accounting for the low buffering capacity of bicarbonate at the particle surface is critical to assess intestinal dissolution of suspensions of weak ionizable drugs [160,161]. That is why including the concept of surface pH has significantly increased the predictivity of PBBM for BCS class II drugs [162,163]. To the best of our knowledge, the concept of surface pH has not been applied to understand precipitation kinetics for weak bases in the intestinal lumen. However, a deep understanding of the dynamics between precipitation and absorption rates would be key to improving the prediction of poorly soluble weak bases dissolution/absorption.

Beyond standard immediate release and controlled release of drug products, PBBM has also been used to predict in vivo performance of enabling formulations. Examples include amorphous solid dispersions (ASDs) and the impact of molecularly dissolved drugs [164,165], surfactant-based formulations [50], use of precipitation inhibitors [166], orally administered nanocrystals [167], Push-Pull Osmotic Pump Tablets [168], and gastroretentive release systems [169], among several others. Some of these assessments have used virtual bioequivalence (VBE) trials. The latter is a powerful tool not only for guiding drug development but also for supporting biowaivers [170,171] and risks associated with post-approval changes in excipient composition [172] or particle size distribution [173].

Similar to PBPK models, integrated approaches have combined PBBM with other NAMs. The study published by Patros et al. advanced this concept by embedding regression equations derived from chip experiments into PBPK platforms. With this model, a PBBM approach was used to assess the effect of different release kinetics on simulated PK profiles [74]. A different integration strategy was published by Khan et al., who combined AI models with PBPK to develop orodispersible moxifloxacin tablets [174]. The authors used central composite design (CCD) and ANNs to create an AI-based quality by design (QbD), and formulation performance was subsequently assessed in the PBPK. Since formulation aspects and PBPK modeling were combined, this integrated approach can be classified as a PBBM application to support drug development. More recently, integration of AI and PBPK was successfully applied to mechanistically explain the impact of molecular dissolution on the performance of ASDs [175]. Together, these examples demonstrate the power of integrating different NAM approaches to reliably predict the biopharmaceutic performance of formulations. In this regard, it is important to highlight that a reduction in the use of animal and even human experimental models depends on the success of NAMs integration. Therefore, consistency and translatability of key parameters across platforms should be demonstrated. Further successful examples and studies, as well as sound discussion on best practices, are needed for these integrated approaches to reach their maximum potential.

Current status and challenges of PBBM have been reviewed somewhere else [8,158]. Furthermore, recommendations for applying the PBBM approach in regulatory and industrial contexts have been recently published in a collection of papers [176,177,178]. The reader is referred to these excellent articles for further information on the subject.

## 4. The Role of NAMs in Biopharmaceutic Assessment Throughout Drug Development: Current Perspectives and Future Directions

Over the past decades, the field of oral biopharmaceutics has evolved in the way it generates and interprets evidence. What once depended almost entirely on well-established but inherently simplified models has gradually expanded into a landscape enriched by human-relevant technologies capable of supporting decision-making across discovery, development, and regulatory evaluation of pharmaceutical products. Rather than replacing standard models outright, NAMs expand the toolkit, offering improved physiological fidelity, deeper mechanistic insight, a reduced ethical burden, and greater readiness for regulatory decision-making. In other words, they provide the connective tissue between empirical observation and human-centered prediction, marking a decisive step toward a more integrated, predictive, and ethically responsible frameworks for biopharmaceutical evaluation throughout drug development. This section summarizes our views on the subject.

### 4.1. Early Stages: Drug Discovery and Preclinical Stages

The literature reviewed in this manuscript evidenced that no single experimental or computational approach can fully support the diverse decisions required across the drug development process. What truly matters is not choosing “the best” model, but rather orchestrating methods in a sequence where each contributes its specific strengths. Early in development, broad in silico screening (whether based on QSPR relationships or more sophisticated ML and DL models) serves as a rapid filter, identifying compounds with promising physicochemical and ADME profiles long before any biological experiments are required. These predictions naturally lead to streamlined in vitro assays, such as PAMPA and/or Caco-2 systems, which provide an efficient first check on permeability and absorption-related liabilities. Nonetheless, ML and DL algorithms may find further value in forecasting outcomes from PAMPA and/or standard Caco-2 assays. This may be possible too with the recently developed in vitro NAMs, provided a robust training dataset is available. Such values can be afterwards integrated into PBPK models to generate a very early prediction of the compound’s in vivo performance. Even though the advances in this subject allow us to consider this possibility, model prediction needs to be improved such that both in vitro and in vivo confirmation of those results may still be necessary.

From there, questions that demand greater physiological relevance are best addressed with advanced NAMs, including organoid cultures and organ-on-a-chip systems operating under flow, where segment-specific microenvironments, dynamic forces, and human-like transporter and metabolic activity begin to close the long-standing gap between preclinical models and human outcomes. Only when absolutely necessary (typically in cases where no in vitro or in silico method can capture a critical aspect of systemic response) could targeted animal in vivo studies still play a fundamental role, functioning as a confirmatory rather than exploratory step. Moreover, integration with PBPK and PBBM models could smooth the transition between different stages of drug development. This concept was nicely demonstrated by Patros et al., who were able to integrate results from OoC experiments into PBPK platforms, enabling accurate simulation of plasma profiles for diverse compounds and formulations [74]. Therefore, organoids and organ-on-a-chip platforms supply high-quality permeability and metabolic data that allow these mechanistic models to operate with far less empirical “tuning,” supporting bottom-up simulations. Hence, we believe that early stages could greatly benefit from integrating different NAMs, thus providing holistic predictions to guide drug development decisions (Table 1).

It is clear that in vitro NAMs methods can be extremely advantageous in replacing ex vivo models that use excised animal tissue as a permeability barrier. However, when reshaping the use of animal models in drug development and regulatory submissions, two complementary trajectories for implementing NAMs can be foreseen. One follows a more traditional, incremental approach, in which NAMs are used primarily to emulate the information historically obtained from animal studies. In this framework, organoids, an organ-on-a-chip platforms, and mechanistic computational models serve to refine hypotheses while reducing the number of animal experiments required. This ensures that by the time an in vivo study is conducted, it functions mainly as a confirmatory gate rather than a primary source of evidence. This approach allows the reintegration of collected data to finetune models and their underlying assumptions. Even though models revised in this manuscript mainly resembled human physiology, NAMs may also be used to predict outcomes of experiments performed in preclinical animal models. For instance, preclinical formulation development has been achieved by a strategy that uses a PBPK model to guide a dog study [179]. More recently, rat and dog intestinal organoids have been developed and characterized for their disposition and metabolic capacities in comparison to human organoids. The evidence with these in vitro NAMs endorsed their potential for pre-clinical toxicity assessment for early identification of toxicity/safety issues before running animal testing [180,181]. Nevertheless, the development of canine colon organoids to assess drug permeability demonstrates that the scope of these models should not be limited to only disposition/metabolism prediction [182].

Alongside this conservative strategy, a far more ambitious pathway is beginning to emerge, where animal experimentation might be altogether bypassed. Here, the evidentiary foundation is built from convergent data streams: physiologically relevant OoC and organoid models, integrated PBPK or PBBM simulations, and increasingly sophisticated AI models that together form a cohesive, human-centered argument for safety and performance. Recent policy shifts signal greater openness of regulators to such fully non-animal approaches. Even though this approach may signify a much cheaper and faster drug development process, its acceptance must be hinged on the accumulation of strong validation exemplars and the establishment of harmonized performance standards. Hence, it is essential to acknowledge both pathways and to recognize that the appropriate balance between them may strongly depend on the nature of the molecule under development, the specific regulatory question (whether exploratory or pivotal), and the sponsor’s overall risk posture.

### 4.2. Late Stages: From Formulation to Post-Approval

When it comes to late stages of drug development, it seems that NAMs are much more well-established. This is probably because of the significant amount of evidence that is typically collected at this stage. A couple of examples of the real-world application of NAMs to waive animal testing in regulatory submissions have been reported, mainly for assessing safety (toxicity assessment) in cosmetic and pharmaceutical industries [3,183,184]. However, some barriers still need to be overcome before reaching regulatory implementation of NAMs as a full replacement of in vivo testing. These barriers include the comfort with the *status quo*, limited acceptance of NAM-generated data, and a narrow regulatory framework, particularly in terms of guidelines and validations [3]. Accordingly, the Next-Generation Risk Assessment (NGRA) concept has been implemented, where NAMs are used in hypothesis testing for supporting regulatory submissions [185]. Days before submitting this manuscript, the FDA published a draft guidance on “General Considerations for the Use of New Approach Methodologies in Drug Development” [12]. Even though this draft addresses important validation issues, such as context of use, human biological relevance, technical characterization, and fit-for-purpose, it only provides general guidelines from a pharmacology/toxicology background rather than from biopharmaceutics and/or PK characterization.

**Figure 5:**
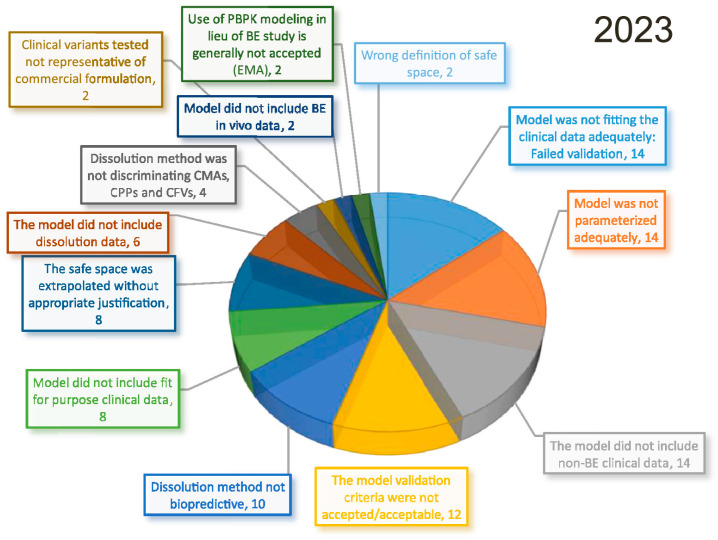
What were the main reasons for the PBBM rejection? Data are expressed as a percentage of the total rejection reasons. Taken from [178].

For biopharmaceutical submissions, PBBM models are currently the most widely used NAM that can waive some in vivo testing. Applications of PBBM have included setting drug product dissolution and particle size specifications, post-approval changes of formulation composition, and manufacturing. More recently, their scope has been expanded towards supporting biowaivers, bridging formulations and justifying dissolution dissimilarities [178]. In recent years, regulatory acceptance of PBBM has increased across agencies, as it was demonstrated by the Canadian agency (Health Canada), where the number of PBBM submissions reviewed and completed was doubled between the period 2021–2022 and 2022–2023 [177]. Further, the same article reported that the FDA has received nearly 50 submissions using PBBM for post-approval changes, where 48% was deemed acceptable. In spite of these advances, it seems that the pharmaceutical industry is still not fully leveraging the potential of PBBM. In fact, a survey revealed that the most common reasons for not using PBBM in regulatory submissions were the “non-familiarity with PBBM” and “business risk due to unknown PBBM acceptance” [177]. This paucity in leveraging PBBM in regulatory submissions is even greater in regions with limited (or non-existing) regulatory guidelines on modeling and simulations [186]. In fact, most of the regulatory submissions with PBBM get rejected because of validation issues (Figure 5), which further supports the role of regulators in providing guidelines. It is important to acknowledge that most PBBM applications considered simulating healthy human physiology, as they intend to mimic traditional bioavailability trials. However, pathological conditions, especially in the GI tract, could affect drug (product) dissolution/absorption. Two reviews have already been published on these interactions [187,188].

Another application of mechanistic modeling comes to support the potential effect of oral absorption-mediated drug interactions on drug product performance. PBPK and PBBM models have been used to simulate either physiological or pathological conditions, including food effect, elevated gastric pH (i.e., coadministration with acid-reducing agents), and altered gastric emptying time. Regulatory submissions for these cases have been submitted and even accepted, resulting in shaping drug product labeling [150]. Similarly, PBPK models can be especially valuable when addressing drug disposition in different populations [189,190]. These tools can be used to guide the development of products with different dose strengths, therefore targeting special populations. In pediatrics, older adults, and genetically diverse groups, differences in transporter abundance, enzyme maturation, intestinal physiology, and systemic handling of drugs can render naïve allometric scaling unreliable, sometimes substantially so. By integrating data generated from NAMs (i.e., permeability measurements from organ-on-a-chip systems), PBPK models can provide a more faithful representation of drug product performance, thus enabling an additional source for supporting drug product labeling.

### 4.3. Biopharmaceutic Classification: Standard Models vs. NAMs

Standard models, particularly for permeability, have been widely used ever since for BCS classification purposes (Section 2). However, the literature reviewed in this article reveals the potential of NAMs in obtaining more physiologically relevant models with presumably higher predictability. From this framework, it naturally raises the question of whether these classical models should be replaced by the NAMs. Here, we still acknowledge the great value of standard permeability methodologies, such as static cell cultures (i.e., epithelial cell model that has successfully demonstrated its suitability (ICH M9 guideline)) for BCS classification, as they have been robustly applied in the past decades with wide regulatory reception. This acceptance is based on the sound physicochemical principles applied to the development of the BCS [191], as well as the fact that BCS-based biowaivers might be understood as a risk assessment-type of framework, where the test product must demonstrate that its in vivo performance would be similar to the reference, even under the worst-case scenario [192]. This means that the BCS framework does not pursue accurate predictions, but it seeks tools with the capacity of detecting potentially relevant differences between products. Regulators overall align well with this type of framework, as they must weigh the patient risk in every regulatory decision.

One possibility that has only been slightly explored is the role of in vitro NAMs assessing the effect of excipients. The recent literature has discussed the oversensitivity of static models to this end [17]. In fact, experimental approaches that include an internal standard have not always proven to control said oversensitivity [156]. Even though statistical considerations may be applied to balance the significance versus relevance of excipient effect on static cultures [156,157], in vitro OoC systems may be helpful, providing a more physiologically relevant experimental setup to answer that question [93], provided the method has demonstrated its suitability [92]. This possibility may enable not only functional testing of the effect of excipients at different stages of drug development but also the conduction of meaningful comparative permeability experiments between reference and test formulations. This type of experiment may be highly important to rationally design bioequivalent generics when the Q1/Q2 compositions of the reference formulation are not known.

Beyond methodological performance, the suitability of NAMs for biopharmaceutic classification and regulatory decision-making must be interpreted in light of both physiological variability and the context of use. Human oral absorption is inherently variable, influenced by inter- and intra-individual differences in intestinal anatomy, transporter and enzyme expression, mucus properties, luminal composition, and gastrointestinal motility [87,187,189]. While advanced NAMs markedly improve physiological relevance compared to static models, they typically represent a defined and controlled biological state, most often derived from healthy adult tissue. As a result, extrapolation to diverse populations, disease conditions, or extreme physiological scenarios requires careful justification and, in many cases, complementary evidence [66,77,87].

Importantly, this limitation is not unique to NAMs. Classical models such as Caco-2 monolayers and animal studies similarly abstract complex physiology into simplified experimental representations [10,17,64]. The key distinction lies in how regulatory frameworks interpret uncertainty. Within BCS-based biowaiver paradigms, conservative and intentionally risk-averse tools have historically been favored because they are designed to detect worst-case scenarios rather than to provide accurate predictions of in vivo performance [7,191,192]. In contrast, NAMs often generate richer, more human-relevant data, but their increased physiological specificity necessitates explicit definition of applicability domains, performance boundaries, and decision context [3,7,12].

From a regulatory perspective, NAM acceptance is therefore best viewed as question-driven rather than model-driven. Agencies such as the FDA and EMA increasingly support NAM use when they are fit-for-purpose, reproducible, and mechanistically informative for the specific regulatory question being addressed. In biopharmaceutics, this often requires integration of NAM outputs into mechanistic frameworks such as PBPK or PBBM, where variability can be explored through sensitivity analyses, virtual populations, and scenario testing. Without such integration, even highly physiological in vitro data may remain difficult to translate into decision-grade evidence [8,12,176,177]. Consequently, NAMs should not be positioned as universal replacements for standard models, but as complementary tools whose regulatory value depends on transparent articulation of limitations, uncertainty, and intended use.

It is important to highlight, though, that the superior physiological relevance offered by NAMs comes at the cost of increased complexity and resource requirements. Platforms such as the human duodenum-on-a-chip integrate biopsy-derived organoids into microfluidic devices, requiring advanced cell culture expertise, ECM coatings, PDMS activation, and continuous flow control [71,74]. Analytical workflows involve high-resolution imaging, barrier integrity monitoring, and LC–MS quantification, making these systems more labor-intensive and expensive than static assays; however, automation and high-throughput chip arrays are emerging to improve scalability and cost-effectiveness [74]. Therefore, for these innovations to move from experimental promise to real-world utility, standardization and regulatory harmonization will be essential. Consensus around chip materials, device geometry, flow regimes, reference compounds, and performance acceptance criteria will help to position NAMs in biopharmaceutical assessment. Finally, since the practical impact of NAMs will also depend on their scalability, advances in 3D printing, automated microfluidics, high-throughput chip arrays, robotics, and cloud-based analytics are valuable, paving the way for NAMs to become realistic tools for large-scale screening and even regulatory submissions.

## 5. Conclusions

Oral biopharmaceutics is evolving toward a landscape where human biologically relevant, mechanistic, and predictive methodologies are critical tools in guiding drug development. The evidence reviewed throughout this manuscript underscores the role of New Approach Methodologies (NAMs), from organoids and organ-on-a-chip systems to machine learning, PBPK, and PBBM methods. Furthermore, current status and future directions were thoroughly discussed, from early stages of drug development to post-approval changes in the drug product lifecycle: While classical biopharmaceutic tools remain scientifically valuable for their conservative nature in regulatory decisions (e.g., BCS-based biowaivers), they embody some limitations that complicate translation to humans. NAMs address these gaps by offering models that better reflect the GI complexity while at the same time using the 3R principle. Likewise, the biorelevance of NAMs makes them ideal candidates for modeling other similarly complex routes of administration. Ultimately, according to the stage of drug development, different NAMs may become more or less relevant. Therefore, choosing the right methodology (or integration of them) plays a key role in the correct integration of NAMs to biopharmaceutic characterizations throughout drug development.

## Figures and Tables

**Table 1:** Recommended New Approach Methodologies (NAMs) across drug-development stages.

Development Stage	Recommended NAMs	Rationale/Typical Use
Early stages (discovery & early preclinical)	- Machine Learning/Deep Learning (ML/DL) - QSPR based in silico screening - PBPK (early predictions) - Organoids (initial absorption & metabolism insights) - Organ-on-a-chip (OoC)	Rapid filtering of candidates, early ADME liability identification, high throughput predictions, early mechanistic understanding before wet lab investment.
Preclinical (mechanistic & translational studies)	- Organoids (transporters, metabolism, disease models) - Organ-on-a-chip (dynamic permeability, flow dependent behavior) - PBPK (mechanistic absorption modeling, transporter contributions) - ML/DL (predicting permeability, bioavailability, formulation attributes)	Human-relevant data for permeability and metabolism; refinement or replacement of animal studies; integration of in vitro NAMs into PBPK for improved predictivity of bioavailability.
Late stages (formulation optimization, regulatory preparation)	- Organ-on-a-chip (comparative permeability, excipient influence) - Organoids (metabolism, special populations) - PBBM (dissolution safe space, dissolution spec setting, tunning drug release kinetics)	Supports formulation bridging, biowaivers, establishing clinically relevant dissolution specifications, and predicting food/pH effects.
Post approval (lifecycle management)	- Organ-on-a-chip (evaluating excipient or minor formulation changes) - PBBM (assessment of post-approval changes, particle size variations, excipient modifications)	Regulatory support for SUPAC/post-approval changes, virtual BE, risk assessment without new in vivo studies.

## Data Availability

No new data were created or analyzed in this study.

## Abbreviations

The following abbreviations are used in this manuscript:

ACAT	Advanced Compartmental Absorption and Transit Model;
ADME	Absorption, Distribution, Metabolism, and Excretion;
ANN	Artificial Neural Network;
ASD	Amorphous Solid Dispersion;
BCRP	Breast Cancer Resistance Protein;
BCS	Biopharmaceutics Classification System;
BSA	Bovine Serum Albumin;
Caco-2	Human Colon Carcinoma Cell Line;
CC BY	Creative Commons Attribution License;
CLint	Intrinsic Clearance;
CYP	Cytochrome P450;
DL	Deep Learning;
ECM	Extracellular Matrix;
EMA	European Medicines Agency;
Fa	Fraction Absorbed;
FaSSIF	Fasted-State Simulated Intestinal Fluid;
FDA	U.S. Food and Drug Administration;
FeSSIF	Fed-State Simulated Intestinal Fluid;
F	Oral Bioavailability;
fu	Fraction Unbound;
GNN	Graph Neural Network;
GoC	Gut-on-a-Chip;
HIF	Human Intestinal Fluid;
ICH	International Council for Harmonisation;
IDAS2	In-Vitro Dissolution Absorption System 2;
IEBC	Intestinal Explant Barrier Chip;
IND	Investigational New Drug;
ML	Machine Learning;
MDR1	Multidrug Resistance Protein 1;
MDCK	Madin-Darby Canine Kidney Cells;
NGRA	Next-Generation Risk Assessment;
OoC	Organ-on-a-Chip;
Papp	Apparent Permeability;
PBPK	Physiologically Based Pharmacokinetic;
PBBM	Physiologically Based Biopharmaceutics Model;
PDMS	Polydimethylsiloxane;
Peff	Effective Permeability;
PEPT1	Peptide Transporter 1;
P-gp	P-glycoprotein;
PDO	Patient-Derived Organoid;
QbD	Quality by Design;
QC	Quality Control;
QSPR	Quantitative Structure–Property Relationship;
SPIP	Single-Pass Intestinal Perfusion;
VBE	Virtual Bioequivalence;
Vd	Volume of Distribution.
